# Nurturing care as a critical buffer against climate change impacts on child development

**DOI:** 10.1371/journal.pgph.0004441

**Published:** 2025-04-08

**Authors:** Jorge Cuartas, Francis Vergunst

**Affiliations:** 1 Department of Applied Psychology, New York University, New York, United States of America; 2 Centro de Estudios sobre Seguridad y Drogas (CESED), Universidad de los Andes, Bogotá, Colombia; 3 Department of Special Needs Education, University of Oslo, Oslo, Norway; PLOS: Public Library of Science, UNITED STATES OF AMERICA

## Abstract

We examine empirical and conceptual considerations related to the role of nurturing care for protecting human capital formation in the context of climate change.

Climate change is a pressing global challenge. Heatwaves, wildfires, storms, and floods are becoming more frequent and severe, and their direct impact and aftermath can have long-lasting negative effects on employment, education, healthcare, and access to essential services. Children are particularly vulnerable to these harms due to their developmental immaturity and limited capacity to mitigate and avoid risks [1,2]. Consequently, parents and other adult primary caregivers – such as grandparents, relatives, and foster parents (hereafter “caregivers”) – provide the primary buffer between climate hazards and adverse developmental outcomes. They do this through *nurturing care*, defined as the provision of stable environments that promote children’s health and nutrition, safety and security, opportunities for learning, and emotionally supportive relationships [3]. Despite the central role of nurturing care for children’s life outcomes, it rarely appears in climate change research and policy discourse.

## Nurturing care promotes and protects child development

Children’s need for nurturing care in order to flourish is enshrined in global policy tools like the Nurturing Care Framework [4]. Nurturing care promotes the development of foundational cognitive, social, emotional, language, motor, and executive function skills that predict lifelong learning, health, and adaptive behaviors central to human capital formation [3]. These benefits are well-documented for children living in poverty, forced displacement, conflict zones, and other emergency settings, indicating that nurturing care also promotes the development of coping mechanisms to overcome early hardship [3]. Despite this, fewer than one in four young children living in low- and- middle-income countries (LMICs) receive minimally adequate nurturing care, which hampers individual development and societal progress and threatens global policy objectives such as the Sustainable Development Goals - SDGs [5,6]. Climate change will likely exacerbate these challenges further.

**Fig 1:**
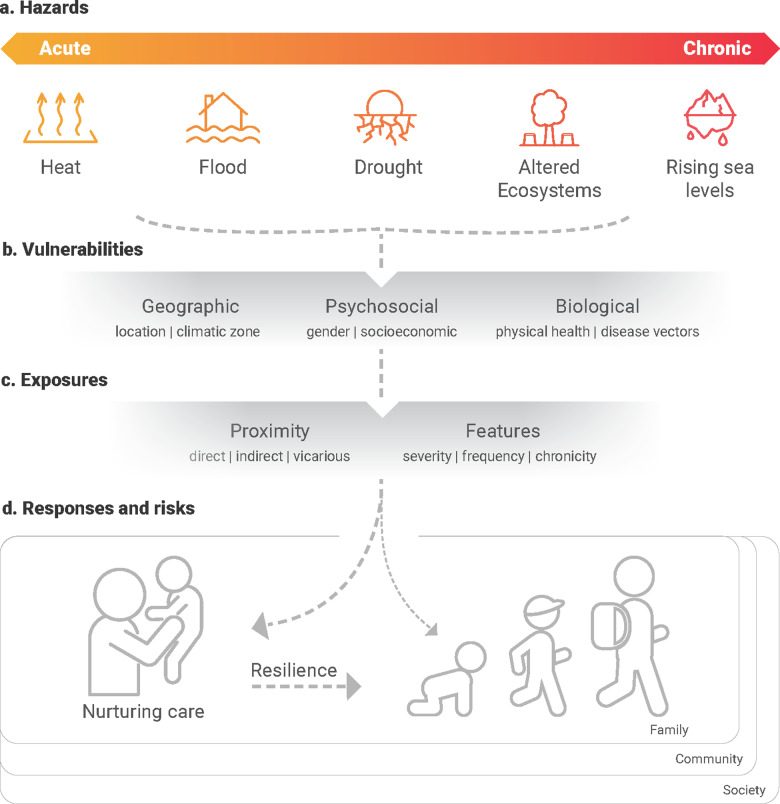
A schematic diagram linking climate-related hazards to adverse child and adolescent developmental outcomes, mediated and moderated by nurturing care. Climate-related hazards (a) are becoming more frequent, severe and long lasting with climate change, and existing geographic, psychosocial, and biological vulnerabilities (b) moderate the risk of exposures (c). Exposures create risks to the caregiver and child, resulting in potential impacts on the child’s development (d). Importantly, these impacts are primarily mediated and moderated by the provision or nurturing care, which depends on the physical, psychological, social, and material resources of the caregiver(s), and the responsiveness and support structures available at the family, community and societal levels.

There is now extensive evidence that climate change is already harming the healthy development of children and adolescents in regions across the world [2,7]. Climate change poses direct and indirect risks to children’s healthy physiological, psychosocial, and neurocognitive development through multiple acute and chronic hazards, such as tropical storms, floods, heatwaves, droughts, altered ecosystems, and sea level rise (Fig 1). When these impacts occur early in life, they can cascade with additive, interactive, and cumulative effects on education, economic participation, and long-term health and wellbeing [1].

## Links between climate change and nurturing care

Evidence from prior crises, such as the COVID-19 pandemic, shows that contextual stressors can compromise caregivers’ ability to provide nurturing care by undermining health, wellbeing, and available material, cognitive, social, and emotional resources. In the context of climate change, extreme weather events can damage and destroy infrastructure, disrupt access to essential services, food and water, and lead to school closures, family separation, and forced displacement – all of which make it more difficult for caregivers to provide for children’s basic needs. Reviews of the evidence indicate robust associations between exposure to disasters and extreme weather events and increased risk of parenting stress and mental health problems such as PTSD, depression, anxiety, substance use, and suicidal behaviors [8]. This mental health burden, in turn, can lead to negative caregiver behaviors, including heightened risk for child physical punishment, reduced learning opportunities, and adverse social and emotional development [8–10].

Caregiving is practically and psychologically demanding. The experience of losing a home or livelihood to flooding, for example, not only undermines material resources but also key cognitive and psychosocial resources critical to providing a nurturing care. It makes it harder for caregivers to regulate their attention and behavioral responses, and raises the risk of inadequate supervision, diminished responsiveness, impaired conflict resolution skills, violence against children, and intimate partner violence [11]. In this way, climate change erodes caregivers’ capacity to respond and adapt to children’s needs and thus provide them with the nurturing care critical for their development, especially when resources are already stretched.

Climate change impacts on caregivers are inequitably distributed. Within nations, low-income families and communities affected by armed conflict, forced displacement, and other adversities are often more vulnerable to exposure to climate related hazards, such as heat stress and flooding, while also having fewer resources to adapt and respond to these stressors. Inequities between rich and poor nations can be even starker: around 89% of the world’s children live in LMICs, which are overwhelmingly located in regions identified as being most vulnerable to climate change [2,12]. As a result, hundreds of millions of caregivers in these regions must contend with already-scarce goods and services in contexts further strained by climate hazards – thus amplifying the risk that caregiving capacity, and therefore child development, will be compromised.

The risks for women are especially acute. Across the world, women provide the bulk of nurturing care and are disproportionately vulnerable to climate-related impacts. For instance, due to persistent discriminatory gender-based cultural norms and practices as well as economic and political barriers [13], girls and women often have more limited access to financial resources, employment opportunities, and property ownership, making it more difficult to independently respond and adapt to climate-related hazards. In some LMICs, they are also frequently dependent on low or unpaid outdoor labor such as water and fuel collection, which directly exposes them to weather-related hazards such as heat stress. Similarly, in the aftermath of acute weather disasters, displaced women are disproportionately vulnerable to violence, exploitation, and trafficking, which present immediate risks to the women themselves and their capacity for care provision. More broadly, women are also commonly under-represented in decision-making processes at the household, community, and policy-making levels, meaning their voices are not heard on issues like climate change adaptation, including those relevant to early care provision.

## Implications for research and practice

Caregivers provide the first and primary buffer against climate-related hazards, including mitigating and responding to impacts. More must be done to understand their unique vulnerabilities. Further conceptual and empirical work are needed to: 1) clarify the domains of nurturing care that are most vulnerable to disruption through exposure to acute and chronic climate-related hazards; 2) identify hazards that pose the most significant simultaneous risks to children’s development and caregivers’ resources through additive, interactive or cumulative effects across the early life course; 3) determine how existing evidence-based programs and policies aimed at promoting nurturing care may need adaptation for climate-affected contexts, and 4) develop gender-sensitive approaches that support caregivers’ mental health and wellbeing so that they can help children to better understand and navigate the psychological consequences of living with climate change. Crucially, these efforts must recognize that LMICs are not a monolith. They encompass a vast range of cultural, geographical, and political contexts – implying that effective strategies for nurturing care will differ across regions and must be informed by community-level needs, resources, and voices.

Climate change is no longer a future possibility but a current reality affecting caregivers and children across the globe. These impacts undermine children’s health and development directly and indirectly by eroding their core source of protection and support: nurturing care. Fortunately, encouraging examples of effective locally tailored interventions and adaptation are emerging. In Malawi, for instance, village-led early childhood development centers have offered supportive environments for young children in the aftermath of floods and other weather disasters, which help to protect both child development and caregiver wellbeing alike [14]. Likewise, in Bangladesh, community support organizations have paired parental mental health with support with early childhood feeding programs to improve outcomes for caregivers and children following tropical cyclones [15]. Programs like these that combine practical resources with mental health and educational support demonstrate the potential to maintain and strengthen nurturing care in the face of climate-related impacts.

By investing in and scaling up these and other context-specific strategies – and by elevating women’s voices in decision-making processes – we can ensure that caregivers and children, especially those in under-resourced settings, are better equipped not just to survive but to thrive in a rapidly changing world.
